# Antipseudomonal *β*-Lactams Resistance in Iran

**DOI:** 10.1155/2020/8818315

**Published:** 2020-12-16

**Authors:** Mohammad Mahdi Rabiei, Keivan Asadi, Shervin Shokouhi, Mohammad Javad Nasiri, Ilad Alavi Darazam

**Affiliations:** ^1^Clinical Research Development Unit of Loghman Hakim Hospital, Shahid Beheshti University of Medical Sciences, Tehran, Iran; ^2^Infectious Diseases and Tropical Medicine Research Center, Shahid Beheshti University of Medical Sciences, Tehran, Iran; ^3^Department of Medical Microbiology, School of Medicine, Shahid Beheshti University of Medical Sciences, Tehran, Iran

## Abstract

Over the last years, the mortality rate of *Pseudomonas aeruginosa*, which is one of the major reasons for severe infections, has been significantly increasing. This bacterium is highly resistant to many antibiotics, especially carbapenems, thanks to its complicated mechanism by which it can acquire exogenous genes. The purpose of this research is to have a review of empirical studies surveying the *P. aeruginosa* resistance to beta-lactams in Iran in order to investigate the most reliable methods by which the incidence of *P. aeruginosa* infections can be decreased and controlled. We performed a systematic review of all articles published from 2008 until 2018. Studies which did not address *P. aeruginosa* resistance to beta-lactams were excluded from the analysis. Studies with less than 10 cases were also excluded. Studies with more than ten cases, which did not have repetitive information, were taken into account for the final selection; 133 out of 893 articles were chosen. The resistance rate of *P. aeruginosa* among the articles was as follows: more than 72% of studies revealed >50% level of resistance to cefepime, followed by aztreonam (53.2%), ceftazidime (61%), piperacillin/tazobactam (54.5%), meropenem (48.3%), and imipenem (42.4%). The selection of empiric antipseudomonal antibiotics is absolutely uncertain and hazardous, and the risk of clinical failure may be more among cephalosporins and piperacillin-tazobactam as well as aztreonam. The results of this study illustrate that the methods enabling clinics to identify the bacterium resistance pattern and its genetic basis and to have the opportunity of empiric therapies through access to updated local data of antimicrobial susceptibility pattern are the most effective methods. However, the widespread usage of these approaches undoubtedly needs reliable molecular and nucleic acid-based devices, which are both affordable and available.

## 1. Background

*Pseudomonas aeruginosa (P. aeruginosa)* is an extensively drug-resistant (XDR) pathogen emerging in hospitals across the globe. It can cause severe healthcare infections and has high mortality rates [1]. In addition to intrinsic drug-resistance mechanisms, this bacterium is able to acquire exogenous genes resulting in resistance to antibiotics specially carbapenems [2].

It is the most important pathogen causing ventilator-associated pneumonia (VAP) and burn wound infections [3] and is the most frequent and severe cause of chronic respiratory infections in patients suffering from cystic fibrosis (CF) or other chronic underlying diseases such as bronchiectasis and chronic obstructive pulmonary disease (COPD) [4].

Genetically, *P. aeruginosa* has outstanding intrinsic antibiotic-resistance mechanisms including the constitutive (MexAB-OprM) or inducible (MexXY) expression of efflux pumps and the reduced permeability of outer membrane [5]. Acquired resistance is due to the production of beta-lactamase enzymes such as extended spectrum beta-lactamase (ESBL), metallo *β*-lactamases (MBL), and sometimes plasmidic AmpC *β*-lactamases. ESBLs are beta-lactamases that hydrolyze penicillins, cephalosporins, and aztreonam, while MBLs hydrolyze carbapenems and other beta-lactams [6]. The adaptive resistance of *P. aeruginosa* involves the formation of biofilm in the lungs of infected patients where it favours as a diffusion barrier for antibiotic access to the bacterial cells [7].

Treatment of *P. aeruginosa*'s infections has become a great challenge due to the ability of this bacterium to resist many of the currently available antibiotics. The World Health Organization (WHO) has recently listed carbapenem-resistant *P. aeruginosa* as one of three bacterial species for whom a critical need for the development of new antibiotics to treat infections is essential [8].

In the present systematic review, we analyzed the *P.aeruginosa* resistance pattern in Iran as coming from the studies published over the last ten years.

## 2. Materials and Methods

### 2.1. Literature Search

According to the PRISMA guidelines, a literature review was carried out by several databases, including Web of Sciences, Scopus, PubMed, and Google Scholar, and the Persian scientific search engines MagIran, IranMedex, and Scientific Information Database (SID). Medical terms including “*Pseudomonas aeruginosa*,” “*P. aeruginosa*,” “Drug resistance, microbial,” and “Beta-lactams” and other keywords such as “Iran,” “antimicrobial resistance,” “bacterial resistance,” “microbial resistance,” and “drug resistance” were searched either separately or in combination. In addition to articles retrieved with this method, the reference lists were also reviewed. All articles written in English or Persian (published in 2008–2018) were retrieved.

### 2.2. Inclusion Criteria

Studies with relevant and unrepetitive information were included in the final analysis. Studies that provided data on the antibiotic resistance in clinical isolates *of P. aeruginosa* and/or described the mechanisms of beta-lactams resistance in detail were selected. In addition, the bibliography of each article was reviewed to identify additional relevant articles. Among English and Persian articles, studies were included in the current systematic review if they were (1) full text, (2) original research, (3) susceptibility data for at least one antipseudomonal drug, and (4) resistance determined according to Clinical and Laboratory Standards Institute (CLSI) Guideline.

### 2.3. Exclusion Criteria

Firstly, studies which did not address *P. aeruginosa* resistance to beta-lactams were excluded from the analysis. Studies with at least one of the following aspects were excluded: (1) studies that were not relevant with environmental data, (2) studies with languages other than English or Persian, (3) studies with less than ten cases (4) review articles, (5) case reports, (6) editorials, and (7) articles with no eligible data.

### 2.4. Data Collection

Articles with the following features were also excluded: (1) articles published both in English and Persian (in these cases, the article published with more detailed results was chosen) and (2) duplicated publications.

For each study, the following information was extracted: author's name, published year, study period, study design, sample size, type of infection, sites of infection, number of *P. aeruginosa* tested, proportion of beta-lactams resistance, the method of diagnosis of beta-lactams resistance, and the mechanisms of beta-lactams resistance. Literature identification and data extraction were performed by two researchers independently.

Quality assessment of methodological sections and results of included articles was performed by use of STROBE checklist (http://www.equator-network.org).

## 3. Results

**Figure 1:**
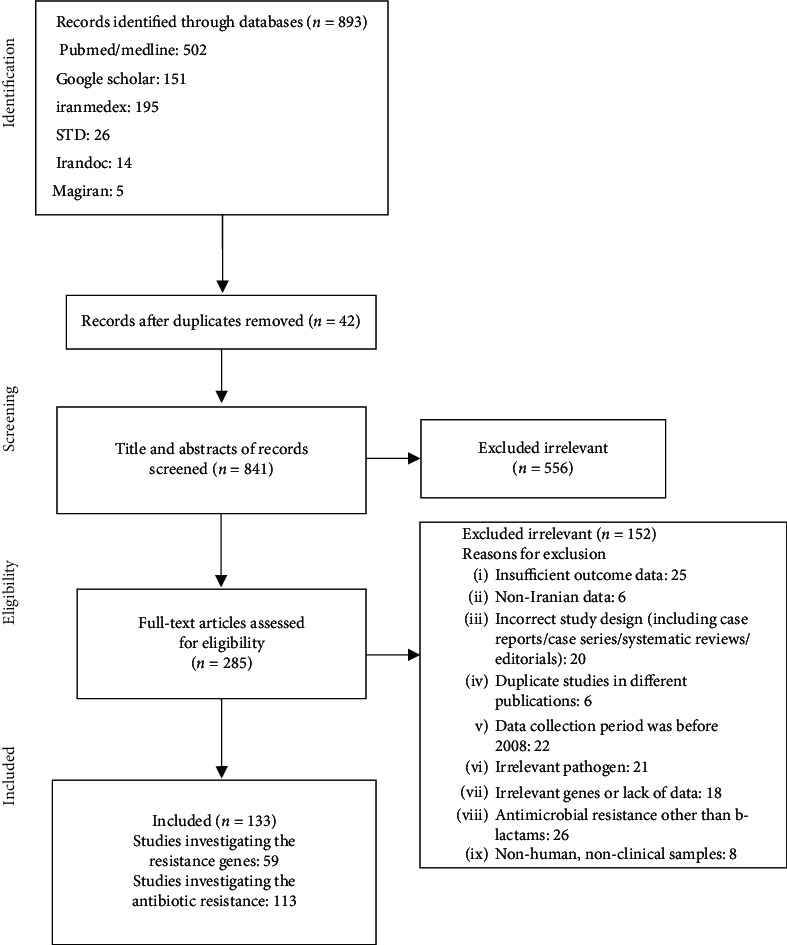
Flow chart of study selection for inclusion in the systematic review and meta-analysis.

Figure 1 shows the steps we followed to select the relevant studies. According to the PRISMA guideline, we initially identified 893 potentially relevant studies from databases. Exclusion was based on title not relevant and duplicates, and 133 articles were retrieved for detailed full-text evaluation. A majority of studies (44 articles) were conducted in Tehran, followed by Isfahan (16), Zahedan, Tabriz, Rasht, Arak, Bandar Abbas, Mashhad, Shiraz, Birjand, Hamadan, Kashan, Kermanshah, Shahr-e kord, and Yazd. Two reviewers (MMR and IAD) independently performed the systematic search by studies selection and data extraction from included studies. Any discrepancies were resolved through consensus discussion. A total of 133 cases were retrieved encompassing. In total, 11,600 cases had been analyzed in these studies (sample size range: 12–573). The mean number of cases in the retrieved articles was 93.8 ± 79.4.

The resistance rate of *P. aeruginosa* among the articles was as follows: more than 72% of studies revealed >50% level of resistance to cefepime, followed by aztreonam (53.2% of articles), ceftazidime (61%), piperacillin/tazobactam (54.5%), meropenem (48.3%), and imipenem (42.4%).

Some studies surveyed few genes of antibiotic resistance and were conducted without specific targeting, whereas few of them specifically focused on the evaluation of related genes. Detailed data regarding all aforementioned issues are shown in Tables 1 and 2.

## 4. Discussion

Carbapenems are more frequently used in clinical settings, and only meropenem and imipenem are available in Iran. Regarding meropenem, in only 10% of studies, the rate of resistance was less than 10%, and in 48.3% of the studies, the rate of resistance was declared more than 50%. In 10.3% of studies, the rate of resistance was estimated more than 90%. Considering imipenem, the mentioned studies highlighted the following points: the rate of resistance more than 50% was estimated in 42.4% of studies, resistance more than 90% was found in about 6.1% of studies, and resistance rate less than 10% was present in only 14.1% of studies. The resistance pattern of doripenem was evaluated in a few studies, and only one out of 7 articles considered resistance rate more than 95% and one study considered less than 10%.

The majority of studies showed the similar resistance pattern considering imipenem and meropenem. Only few studies reported significant differences between the resistance rate of imipenem and meropenem [5, 9–12]. In one of these studies, the reported difference in resistance was about 40% (meropenem: 88.4% and imipenem: 48.4%) with samples coming from hospitalized patients. The samples of this study were not significantly different compared with other articles. Also, the authors did not declare any explanation about these findings [12].

Another article, whose main objective was the environmental biofilms in clinical settings, reported the difference of about 40% (imipenem 30% and meropenem 70%), and the main objective of this article was study on the clinical cases and environmental biofilms. In this study, only clinical cases have been included. The resistance rate of piperacillin/tazobactam was similar to imipenem within 24%, and the resistance rate of cefepime (64%) and aztreonam (60%) was similar to meropenem (64% and 60%). The authors of this study studied only two genes (TEM and SHV) in varied clinical samples that were reported positive 92% for TEM and 16% for SHV [5].

Another study about children reported a noticeable difference rate of resistance between imipenem (15.5%) and meropenem and aztreonam (about 32%). Most samples were clinical urine specimens. Only MBL-associated genes including IMP and VIM were evaluated, 3.3% and 0%, respectively [11].

Among the fourth generation cephalosporins, such as cefepime, only less than 3.7% of studies reported a rate of resistance less than 10% [2, 13], whereas in more than 20% of articles, the rate of resistance considered more than 90% [14–24]. More than 72% of studies revealed >50% level of resistance.

Regarding the third generation cephalosporins active on *P. aeruginosa* such as ceftazidime, 61% of studies found a resistance rate of more than 50%. Moreover, the rate of resistance was calculated more than 90% in 9.6% of studies [22, 24–28].

The rate of resistance in the majority of articles concerning cefepime and ceftazidime were similar, and only a few of them revealed significant difference. In one study, all the samples were taken from burn wounds. The significant number of samples had carbapenemase (50.9%), and most of them were with positive VIM gene. Furthermore, a majority of samples were AmpC overproducer, only one of them was associated with oprD, and 56.5% were oprD downregulated [29].

In another study, the resistance rate of cefepime and ceftazidime was high (61% and 42.8%, respectively), and only genetic evaluation was taken within MBL positive cases. Thus, there is no detailed information regarding cefepime and ceftazidime [9]. Another article reported the resistance of ceftazidime less than carbapenems and cefepime. Around 58.25% of samples were ESBL-positive, and the resistance rate of cefepime was similar in both ESBL-positive and ESBL-negative cases. However, there was a significant difference about ceftazidime resistance rate between ESBL-positive (66.7%) and ESBL-negative cases (28.3%). All of the ESBL-positive cases, which were resistant to cefepime, contained *bla*_PER-1_ and VEB genes, and regarding ceftazidime, the ratio was estimated to be 75% and 100%, respectively [20].

In a study with significant difference in the resistance pattern between cefepime (100%) and ceftazidime (35%), various clinical samples were analyzed, and the aim was to evaluate quorum sensing without data about the resistance genes associated with two aforementioned antibiotics [18].

Because of lacking data regarding genetic patterns of antimicrobial resistance, the studies of Amini et al. and Poorabbas et al. could not be evaluated [17, 30].

Regarding piperacillin/tazobactam, a resistance rate of more than 50% was registered in 54.5% of studies, and in about 10% of studies, the rate of resistance considered more than 90%. Only three studies (6.8%) reported a rate of resistance less than 10% [31–33].

Aztreonam was not available in Iran. In this review, 46 studies evaluated the resistance rate of this monobactam and revealed about 53.2% of articles considered more than 50% resistance rate. Only in four articles, the rate of resistance was estimated less than 10%.

Various OXA-type genes were studied in eight studies (two studies studied all the different types of OXA genes). *bla*_OXA-1_, *bla*_OXA-2_, *bla*_OXA-3_, *bla*_OXA-4_, *bla*_OXA-10_, and *bla*_OXA-48_ were the reported types. About *bla*_OXA-10_, which was studied in eight studies, in six of them, the frequency rate was ≥50% and in two studies, 0% and 3% [22, 23, 34–39]. *bla*_OXA-48_ was studied in one study, and the frequency rate was 0%.

The evaluation of AmpC was performed in nine studies; of which, four were reported to be 100%, and in five studies, the ratio was higher than 50% [14, 23, 29, 31, 40–44].

There are some common genes associated with metallo-lactamase class, including IMP, VIM, and NDM. The VIM gene was observed in twenty studies, and the incidence of *bla*_VIM-1_ was reported as 9.1–60% and for *bla*_VIM-2_ as 0 to 3.17%. In few studies, the VIM gene was studied without defining the type. In only one of these studies, the positivity rate of VIM gene was above 50%, and in other studies, it was between 0% and 47%. The frequency rate of imp gene was thoroughly various, between 0% and 100% [4, 9, 11, 12, 16, 21, 23, 25, 45–55].

The limitations of the present study are due to the differences between setting, sampling, and size of studies as well as the methods to detect antimicrobial resistance and genetic evaluation. Since the collected data could not be reanalyzed, we had to exclude the majority of the studies, thus missing important data.

According to these issues, the authors of the present systematic review decided to evaluate all the related studies one by one and consider all the detailed data among these articles.

## 5. Conclusions

This systematic review revealed that resistance rate of *P. aeruginosa* against most available and utilized antimicrobials is high among hospitalized patients. More than 50% resistance rate was highest in ceftizoxime and cefepime and lowest in carbapenems (less than 50%), and the least is related to imipenem (42.4%). It means the selection of empiric antipseudomonal antibiotics is absolutely uncertain and hazardous, and the risk of clinical failure may be more among cephalosporins and piperacillin-tazobactam as well as aztreonam.

Because of lacking data, evidence-based description of genetic basis of the diversity is not conceivable; however, the high rate of AmpC production and positivity of OXA-type genes suggests caution in selecting antibiotics in the clinical setting.

The most reliable approach is a rapid detection of resistance pattern, its genetic basis, as well as the updated local data of antimicrobial susceptibility pattern for empiric therapies. To use this method, clinicians need affordable rapid molecular and nucleic acids-based devices to be not expensive and available.

## Figures and Tables

**Table 1:** The percentage of antimicrobial resistance.

Antibiotics	Percent of articles reporting high resistance rate (>50%)^∗∗^
Imipenem	42.4
Meropenem	48.3
Aztreonam	53.2
Cefepime	72
Ceftazidime	61
Piperacillin/tazobactam	54.5

^∗∗^High resistance frequency means more than 50%.

**Table 2:** Mechanisms of *β*-lactams resistance (except Ambler class A-associated genes).

Resistance genes	Number of studies	Number of *Pseudomonas* evaluated for resistance genes	Number of genes responsible for beta-lactam resistance
ESBL encoding genes			
*bla*_OXA_	2	140	34
*bla*_OXA-1_	4	472	79
*bla*_OXA-4_	3	267	34
*bla*_OXA-10_	8	542	329
*bla*_OXA-48_	1	53	0

AmpC encoding			
AmpC ∗	9	610	478

MBL encoding genes			
*bla*_IMP_	11	833	226
*bla*_IMP-1_	7	977	139
*bla*_IMP-2_	2	539	11
*bla*_VIM_	14	797	98
*bla*_VIM-1_	5	916	146
*bla*_VIM-2_	6	730	11
*bla*_NDM_	5	470	0

^∗^One study was associated with oprD, and 56.5% was oprD downregulation.

## Data Availability

The data used to support the study are available within the article.
